# Conditioned associations and economic decision biases

**DOI:** 10.1016/j.neuroimage.2010.06.021

**Published:** 2010-10-15

**Authors:** Marc Guitart-Masip, Deborah Talmi, Ray Dolan

**Affiliations:** a Institute of Cognitive Neuroscience, University College London, London W1CN 4AR, UK; b Wellcome Trust Centre for Neuroimaging, Institute of Neurology, University College London, London WC1N 3BG, UK

**Keywords:** Decision making, Learning, Humans, fMRI, Amygdala

## Abstract

Humans show substantial deviation from rationality during economic decision making under uncertainty. A computational perspective suggests these deviations arise out of an interaction between distinct valuation systems in the brain. Here, we provide behavioural data showing that the incidental presentation of aversive and appetitive conditioned stimuli can alter subjects’ preferences in an economic task, involving a choice between a safe or gamble option. These behavioural effects informed a model-based analysis of a functional magnetic resonance imaging (fMRI) experiment, involving an identical paradigm, where we demonstrate that this conditioned behavioral bias engages the amygdala, a brain structure associated with acquisition and expression of conditioned associations. Our findings suggest that a well known bias in human economic choice can arise from an influence of conditioned associations on goal-directed decision making, consistent with an architecture of choice that invokes distinct decision-making systems.

## Research Highlights

►Aversive and appetitive conditioned stimuli can alter subjects’ preferences in an economic choice task. ►These effects are similar to classical, semantically elicited, framing effects. ►This associative behavioral bias engages the amygdala. ►Economic biases may reflect obligatory engagement of Pavlovian response biases associated with reward and punishment.

## Introduction

The idea that humans are optimal decision makers is challenged by substantial evidence for deviations from rationality during economic decision making (Kahneman and Tversky, 1979). Across a variety of contexts, preferences can depend on seemingly arbitrary factors irrelevant to decision outcomes (Kahneman et al., 1991; Tversky and Kahneman, 1981, 1986). The observation that equivalent biases are observed in non-human primates (Chen et al., 2006; Lakshminaryanan et al., 2008), suggests these deviations may, in part, reflect processes and neural substrates that are strongly conserved across phylogeny. It is of interest that functional magnetic resonance imaging (fMRI) studies that have probed the neural underpinnings of one form of decision bias, namely framing, consistently report amygdala activation (De Martino et al., 2006; Roiser et al., 2009), an evolutionary conserved brain structure implicated both in emotional learning in humans and other animals (Phelps and LeDoux, 2005).

Classical explanations of human decision biases often invoke a dual system framework pitting a deliberative rational system against an impulsive myopic affective system (Camerer et al., 2005). However, psychological and neurobiological data point to a more complicated architecture, identifying at least three distinct valuation systems that can compete for control of action. These systems involve Pavlovian, goal directed and a habitual controllers respectively (Dayan, 2008; Seymour and Dolan, 2008). The most basic is a Pavlovian system which learns to predict the value of states and relates these to innate hard-wired behaviour repertoires, such as approach or withdrawal. A more sophisticated goal-directed system uses an explicit model of the environment to choose an appropriate course of action to attain a desired outcome. Finally, the habitual system relies on cached predictions of the expected reward for the set of all possible actions. This type of decision architecture can account for otherwise puzzling behaviour as can arise when there is conflict between Pavlovian and instrumental systems for control of behavior (Dayan et al., 2006). Indeed, hints that such influences may be important in human choice behaviour emerges from previous research showing subjects are more likely to choose an action if a conditioned cue that has previously been paired with the same outcome is present (Bray et al., 2008; Hogarth et al., 2007; Talmi et al., 2008).

In this study our aim was to establish whether incidental influences of conditioned associations, generated through prior conditioning, could elicit a bias in economic decision making similar in form to that seen when outcomes of equivalent expected value are described in terms of a loss or a gain, a well know human bias referred to as ‘framing’. The ‘disease dilemma’ is a popular example of this effect that requires subjects to choose between two scenarios relating to the management of an epidemic (Tversky and Kahneman, 1981). A risky option is fixed, such as ‘Option A has 2/3 chances to save all 600 affected people,’ but a non-risky option is presented in either a positive ‘With option B, 400 people will be saved’ or negative frame ‘With option B, 200 people will die’. A robust finding is that the probability of choosing the risky option increases when the sure option is presented in a negative frame.

**Fig. 1:**
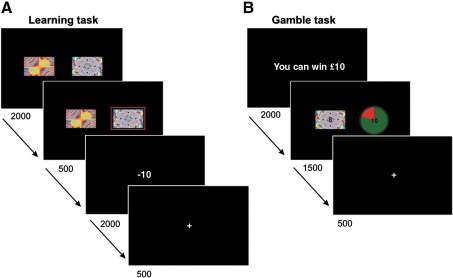
Experimental paradigm. Two tasks were alternated: the instrumental conditioning or learning task and the financial decision making or gamble task. Time is displayed in milliseconds. (A) On learning trials, two colored fractals were presented on the screen; subjects selected one of them, and subsequently observed the outcome. There were 3 pairs: a win, a loss, and neutral pair. In this example, the chosen fractal was associated with losing £10 or losing nothing with 0.8 and 0.2 probabilities, respectively. (B) Each gamble trial began with a message showing the maximum amount of money that could be won in the trial, followed by a choice between a win-all/lose-all gamble (displayed here on the right as a chart pie) and a safe option with equal expected outcomes. The safe option was depicted as a bold number over one a fractal from the learning task: the one associated with 0.8 probability of winning (CSwin), the one associated with 0.8 probability of losing (CSlose), or a neutral fractal (CS-). Subjects were not explicitly told about this manipulation and both tasks were explained as independent of each other. No feedback was provided concerning trial outcomes.

Our experimental paradigm (Fig. 1) alternated two entirely independent tasks involving either an instrumental conditioning (the learning task) or an economic decision-making task (the gamble task). We studied 3 separate cohorts of healthy volunteers (*n* = 20, 16, and 20, respectively) in 3 independent experiments. Experiment 3 was performed while we acquired simultaneous fMRI data with the aim of identifying a neurobiological substrate to the behavioral effects seen in experiment 1 and 2. Both experiment 1 and 2 differ in the time duration of each trial (shorter in experiment 2) and in number of sessions (larger in experiment 2), whereas in experiment 2 and 3 the same protocol was used. This was motivated by a need to adapt and test the protocol from our initial behavioral experiment (experiment 1) to the fMRI environment in order to explore its neurobiological underpinnings. We hypothesized that a bias in decision making during the gamble task engendered by an incidental presentation of conditioned stimuli would lead to amygdala activation, an hypothesis motivated by findings that this brain area is activated in relation to irrational choices in the classical framing effect (De Martino et al., 2006; Roiser et al., 2009).

## Materials and methods

### Subjects

A total of 67 healthy and right handed volunteers were recruited for inclusion in 3 separate experiments: 25 in experiment 1 (13 female, mean age = 25.2 years, SD = 4.2 years), 18 in experiment 2 (7 female, mean age = 24.71 years, SD = 4.95), and 24 in experiment 3 (12, female, mean age = 22.2 years, SD = 3.94). Any subject that failed to show gambling during the task (< 0.5% of their choices) was excluded from analysis (5 subjects from experiment 1, 2 from experiment 2, and 4 from experiment 3). The study was conducted with the approval of the University College London Research Ethics Committee.

### Experimental paradigm

The experiment consisted of two independent alternating tasks, an instrumental conditioning task and a gamble task. An initial learning phase involved an instrumental task with monetary outcomes, similar to that used by Pessiglione et al. (2006), where a pair of colored fractal images was visually displayed on a monitor with subjects being required to select one of them with an explicit goal to maximize their total wins. A total of 3 pairs were used during the entire experiment: a win pair (generated by appetitive conditioning), a lose pair (generated by aversive conditioning), and a neutral pair (always followed by a nil outcome £0). More specifically, each of these pairs was associated with two outcomes: win £10/£0, lose £10/£0, and £0/£0. For the gain pair, the probabilities of winning £10/£0 were 0.8/0.2 for one stimulus and 0.2/0.8 for the other. Similarly, in the lose pair, the probabilities of losing £10/£0 were 0.8/0.2 for one stimulus and 0.2/0.8 for the other. Subjects were told to learn, by trial and error, stimulus-outcome (reward or loss) associations for the win and lose pairs. Thus, on each trial, one of the pairs was randomly presented with the two stimuli displayed right or left from the centre of the screen (see Fig. 1A). The relative position of the stimuli was counterbalanced across trials. Subjects were required to choose the right or left stimulus by pressing a right or left arrow on a keyboard in experiment 1 and 2 or by pressing a right or left key in a MRI-compatible keypad in experiment 3. 2000 ms after the stimuli were presented (3000 ms in experiment 1) the choice was highlighted within a red square for 500 ms. Thereafter, the outcome was displayed on the screen for another 2000 ms, and a fixation cross was displayed for 500 ms before the following trial begun.

The gamble task was similar to the one used to elicit the framing effect (De Martino et al., 2006; Roiser et al., 2009). At the beginning of each trial, subjects were shown a message for 2000 ms indicating the maximum amount of money they could win in the trial. Five amounts of winnings were used in all experiments (£20, £15, £10, £5, £1). Thereafter, subjects were instructed to make a choice between a win-all/lose-all gamble and a safe option with equal expected value (with the exception of catch trials, see below) displayed on the screen for 3000 ms in experiment 1 and for 1500 ms in experiment 2 and 3 (see Fig. 1B). To win the maximum amount of money, participants had to choose the gamble which was represented as a pie chart depicting the probability of winning the gamble in green and the probability of losing the gamble in red. A number in bold font, depicted over the pie chart, indicated the maximum amount of money that could be won on the gamble. Four different probabilities were used in all experiments such that the probability of winning the gamble in a given trial was 0.8, 0.6, 0.4, or 0.2. This contrasted with the safe option which paid a portion of the total amount with probability of 1. The safe option was depicted as a bold number on the other side of the screen and was superimposed over one of the fractals used in the learning task comprising a fractal associated with a high probability of winning (CSwin), one associated with a high probability of losing (CSlose) or one of the neutral fractals (CS-). The CS's were never presented under the gamble option since the experimental question rested on testing whether effects observed in a classical framing task (De Martino et al., 2006; Roiser et al., 2009) could be recreated solely by replacement of the words ‘keep’ and ‘lose’ on the safe option by imposition of conditioned stimuli (CSwin and CSlose) that signaled a win or loss respectively. The CS- condition represented a behavioral control condition to enable us to assess the impact of the learned value, generated by presentation of the CSwin and CSlose, on a decision to opt for the sure or gamble option in the economic task. This exact design pertained to each of the 3 separate experiments.

Subjects were not explicitly told the reasoning behind presentation of fractal stimuli during the economic decision task. In fact, they were told both tasks were independent. Just as in the instrumental conditioning task, subjects choose the option on the right or the left by pressing a right or left arrow on a keyboard in experiment 1 and 2 or by pressing a right or left key in a MRI-compatible keypad in experiment 3. Trials with different CSs, maximum amounts and different probabilities of winning the gamble were randomly intermixed. No feedback concerning trial outcomes was given during the experiment and subjects were told that, at the end of the experiment, three trials of the learning task and three trials of the gamble task would be randomly selected and that they would get paid the selected amount up to a maximum of £45.

Given the equivalence of choices in terms of expected value, we included ‘catch’ trials to ensure that subjects remained engaged in the decision-making task throughout the course of the experiment. In these trials, the expected outcomes for the sure and gamble option were markedly unbalanced: in half of the trials the gamble option had a 0.95 probability of winning and was highly preferable and for the other half of the trials the sure option was preferable since the probability of winning the gamble was only 0.05. As in the rest of the trials, the sure option was also displayed over one of the fractals used in the learning task. As illustrated in Fig. S4, subjects were highly accurate in making optimal decisions in these trials indicating a stable task engagement.

Experiment 1 and 2 were pure behavioral experiments, whereas experiment 3 was performed inside the scanner. In order to get familiarized with the tasks subjects were given practice and any questions that arose were answered. After that, subjects performed several trials of the instrumental conditioning task (learning task): 24 trials of each pair in experiment 1 (7.5 min), and 30 trials of each pair in experiment 2 and 3 (8.5 min). After these initial trials of the learning task, subjects participated in 2 sessions (19.5 min each) of alternating tasks in experiment 1, and 3 sessions (12.7 min each) of alternating task in experiment 2 and 3. Prior to a task block subjects were instructed with the name of the task which was about to begin (learning or gamble task) for 1000 ms. In each session of the alternating task, 10 trials of the gamble task were followed by 9 trials of the learning task (3 of each pair) with a total of 80 gamble trials (20 trials with the CSwins, 20 trials with the CSlose, 20 trials with the CS-, and 20 ‘catch’ trials. The order of the trials was randomized to increase design efficiency. We alternated the two tasks in order to prevented extinction of the association between the CS and win and lose outcomes as subjects performed the separate gamble task.

At the end of the experiment, subjects were debriefed and asked about their strategies when performing the task, their awareness of the presence of the CS from learning task in the gamble task, and whether this manipulation changed their choice preference in the gamble task.

### Behavioral data analysis

The behavioral data were analyzed using the statistic software SPSS. We used a 2 way repeated-measures ANOVA with CS (CSwin; CSlose; CS-) and session (session 1 and session 2 in experiment 1; and session 1, session 2, and session 3 in experiments 2 and 3) as factors on the percentage of trials in which subjects chose the gamble option.

### Computational model

The influence of the CS on gamble preference was observed to change as a function of session in experiment 3. To render our imaging analysis sensitive to this session effect, and capture the neural underpinnings of the behavioral effect expressed in the scanning environment, we generated a parametric regressor of the CS effects on a trial-by-trial basis. We modeled the learning and the gamble tasks separately. The trial by trial estimates of the action-state values (Q) for choosing the different stimuli in the learning task were used to generate biases induced by these stimuli in our modeling of the gamble task. For completeness, we also applied the model to the data of experiment 1 and 2 and show these simulations in supplemental data.

#### Modeling of the learning data

For the learning task data, we fitted a standard reinforcement learning algorithm to each subject's sequence of choices (Sutton and Barto, 1998). We used a basic Q learning algorithm, as this provides a good account of instrumental choice in both humans and primates (O'Doherty, 2004; Pessiglione et al., 2006; Samejima et al., 2005). For each pair of stimuli a and b, the model estimates the expected value of choosing a (*Q*_a_) and choosing b (*Q*_b_), on the basis of individual sequences of choices and outcomes. This *Q* value is essentially the expected reward obtained by taking that particular action (since there are no longer term consequences of these choices). The *Q* values were set at zero before learning, and after every trial *t* > 0 the value of the chosen stimulus (say a) was updated according to the following rules:

$$Q_{\text{a}}\left(t+\text{1}\right)=Q_{\text{a}}\left(t\right)+\alpha *\delta \left(t\right)$$

where *δ*(*t*) is the prediction error:

$$\delta \left(t\right)=R\left(t\right)-Q_{\text{a}}\left(t\right)$$

for *R*(*t*) being the reinforcement obtained as an outcome of choosing a at trial *t*. The reinforcement magnitude *R* was + 10 and −10 for winning and losing £10, and 0 for those outcomes without any monetary consequence. Given the *Q* values, the associated probability of selecting each action (say a) was estimated via the softmax rule:

$$P_{\text{a}}\left(t\right)=\text{exp}\left(\beta *Q_{\text{a}}\left(t\right)\right)/(\text{ exp}\left(\beta *Q_{\text{a}}\left(t\right)+\text{ exp}\left(\beta *Q_{\text{b}}\left(t\right)\right)\right)$$

This is a standard stochastic decision rule that calculates the probability of taking one out of a set of actions according to their associated values which has been shown to account for choice behavior in similar paradigms (Daw et al., 2006; Pessiglione et al., 2006).

**Fig. 2:**
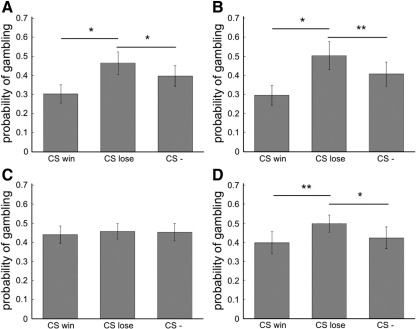
Behavioral results. This figure displays mean percentage of trials in which subjects chose the gamble option for each of the CS's displayed under the safe option. Error bars indicate SEM. Post hoc comparisons were implemented by means of repeated-measures *t*-test: * *P* < 0.05; **P < 0.005. (A) Outside the scanner, subjects showed an increased preference for the gamble option when the CS loss stimulus was presented with the sure option and a decreased preference when the CSwin was displayed during the whole experiment. (B) Outside the scanner, this effect was stronger during the last session of the experiment. (C) Inside the scanner, the emergence of the behavioral effect was not evident if all sessions were considered together. (D) Inside the scanner, the behavioral effect and became only evident during the third session.

The constants *α*_*i*_ (learning rate) and *β*_*i*_ (temperature) were adjusted individually for each subject (*i*) to maximize the likelihood of the actual observed choices under the model. The mean *α*/*β* parameters for the win and lose pairs, respectively, were 0.2/0.56 and 0.28/0.45 in experiment 1, 0.11/0.76 and 0.29/0.58 in experiment 2, and 0.26/0.6 and 0.23/0.58 in experiment 3. The individual subjects’ parameters are reported in Figs. S7–S9. These mean parameters were used to generate the simulations shown in Fig. 2C and Figs. S5C and S6C. The mean likelihood of the actual choices under the model, across all learning trials, was 0.78 in experiment 1, 0.85 in experiment 2, and 0.83 in experiment 3. As a measure of the quality of the behavioral fit of the computational model we report a pseudo-*R*^2^ statistic (Daw et al., 2006) in Tables S1-S3.

#### Modeling of the gamble data

For the gamble task data, we fitted each subject's sequence of choices using a variation of the softmax rule used in the learning task that accounted for the observed behavioral data. On each trial of the gamble task there were two options, the safe and the gamble option, with equal expected value. According to the softmax rule, equivalence in expected value results in choice indifference. However, each subject showed a different underlying preference for the gamble and the sure option which we modeled as a subject-specific parameter (*λ*_*i*_, for subject *i*) added to the value of the sure option. *λ*_*i*_ would be positive for a risk-averse subject and negative for a risk-seeking subject. Moreover, as observed in the behavioral data, subjects’ preference for the gamble or sure option was modified by the presence of the different CSs, and this effect differed across sessions. To account for these behavioral observations we added a bias value (*Q*_*cs*_) to the expected value of the safe option and weighted it by a second, subject (*i*) and session (*s*)-specific parameter, *ε*_*is*_. *Q*_*cs*_ were obtained on a trial by trial basis by modeling the learning task and were equivalent to the current *Q* value of the CS displayed under the safe option. Note that although subjects reached asymptote in the learning task, the *Q*_cs_ value was not stable throughout the gamble task as they were dependent on choices in the learning task that alternated with the gamble task. *Q*_CS_ value was positive for CSwin and negative for CSlose, thereby increasing or decreasing the value of the sure option, an approach similar to that previously implemented in a model of Pavlovian–instrumental interactions (Dayan et al., 2006).

Since the expected value of both options was equal, we could simplify the softmax rule and reduce the *β* parameter or temperature to the following:

$$P_{\text{sure}}\left(t\right)=\text{exp}(\varepsilon_{\text{is}}*\;Q_{\text{cs}}\left(t\right)+\lambda_{\text{i}})/(\text{exp}(\varepsilon_{\text{is}}*\;Q_{\text{cs}}\left(t\right)\;+\lambda_{\text{i}})+\text{1})$$

and

$$P_{\text{gamble}}\left(t\right)=\text{1 }-\;P_{\text{sure}}\left(t\right)\text{.}$$

We calculated the *λ*_*i*_ for each subject using the mean probability of choosing the sure option in trials where the CS- was displayed under the safe option. Since on these trials *Q* = 0,

$$\lambda_{\text{i}}=\text{log}\left(P_{\text{sure}}/\left(\text{1}-P_{\text{sure}}\right)\right)\text{.}$$

The constant *ε*_*is*_ was the only parameter that was fitted to maximize the likelihood of the actual choices under the model, a parameter that we allowed to take on a different value for each of the modeled sessions. In experiment 1, the mean *λ* was 0.53, and the mean ε was 0.05 and 0.1 for session 1 and 2, respectively. In experiment 2, the mean *λ* was 0.41, and the mean ε was − 0.02, 0.03, and 0.07 for session 1, 2 and 3, respectively. Finally, in experiment 3, the mean *λ* was 0.15, and the mean ε was −0.01, −0.007, and 0.03 for session 1, 2 and 3, respectively. The individual subjects’ epsilon parameters are reported in Figs. S7–S9. Note that for experiment 3 the mean ε parameter only differed from 0 for the last session in line with the emergence of the key behavioral bias (mean ε in session 1 was −0.01, *t*_19_ = 0.7, *p* > 0.1; mean *ε* in session 2 was −0.007, *t*_19_ = 0.75, *p* > 0.1; mean *ε* in session 3 was 0.03, *t*_19_ = 2.9, *p* = 0.009). These parameters were used to generate the simulations shown in Fig. 2C and Figs. S5C and S6C.

The mean likelihood of the actual choices under the model, across all gamble trials, was 0.61 in experiment 1, 0.58 in experiment 2, and 0.57 in experiment 3. As a measure of the quality of the behavioral fit of the computational model we report a pseudo-*R*^2^ statistic (Daw et al., 2006) in Tables S1-S3. We acknowledge that a lack of alternative models with which to compare the model fit precludes any strong conclusion about the computational implementation of this specific model. Moreover, the mean and individual pseudo-*R*^2^ reveals that this is clearly not a good model for the subjects' behavior: in many subjects the pseudo-*R*^2^ is virtually 0 and in some cases it is even slightly negative indicating that a model of random choice can perform better. However, our intention in using this model was solely to capture key features of the influence of the CS's on decision making, namely differences in probability of gambling according to whether a CS displayed under the safe option was associated with wins or loses in the learning task. Moreover, because the model allowed us to construct a parametric regressor of the CS effect on the gamble task in fMRI data analysis, this had the added value of increasing the power of our analysis.

### Image acquisition and analysis

For experiment 3, we performed fMRI on a 3-Tesla Siemens Allegra magnetic resonance scanner (Siemens, Erlangen, Germany) with echo planar imaging (EPI). In the functional session 48 T2*-weighted images per volume (covering whole head) with blood oxygenation level-dependent (BOLD) contrast were obtained (matrix: 64 × 64; 48 oblique axial slices per volume angled at −30° in the antero-posterior axis; spatial resolution: 3 × 3 × 3 mm; TR = 2880 ms; TE = 30 ms). The fMRI acquisition protocol was optimized to reduce susceptibility-induced BOLD sensitivity losses in inferior frontal and temporal lobe regions (Weiskopf et al., 2006). For each subject functional data were acquired in three scanning sessions containing 260 volumes per session. Six additional volumes at the beginning of each series were acquired to allow for steady state magnetization and were subsequently discarded. Anatomical images of each subject's brain were collected using T1 weighted sequences (spatial resolution: 1 × 1 × 1 mm). Additionally, individual field maps were recorded using a double echo FLASH sequence (matrix size = 64 × 64; 64 slices; spatial resolution = 3 × 3 × 3 mm; gap = 1 mm; short TE = 10 ms; long TE = 12.46 ms; TR = 1020 ms) for distortion correction of the acquired EPI images (Weiskopf et al., 2006). Using the ‘FieldMap toolbox’ (Hutton et al., 2002) field maps were estimated from the phase difference between the images acquired at the short and long TE.

Pre-processing included realignment, unwrapping using individual fieldmaps, and finally spatial normalizing to the Montreal Neurology Institute (MNI) space and smoothing with a 4 mm Gaussian kernel. The fMRI time series data were high-pass filtered (cutoff = 128 s) and whitened using an AR(1)-model. For each subject a statistical model was computed by applying a canonical hemodynamic response function (HRF) combined with time and dispersion derivatives (Friston et al., 1998).

At the first level model all sessions were concatenated and the following 4 conditions of interest were modeled as events at the onset of the trial: trials in which subjects chose the sure option where the CSwin was displayed under the safe option (Sure/CSwin), trials in which subjects chose the gamble option where the CSwin was displayed under the safe option (Gamble/CSwin), trials in which subjects chose the sure option where the CSlose was displayed under the safe option (Sure/CSlose), and trials in which subjects chose the gamble option where the CSlose was displayed under the safe option (Gamble/CSlose). As parametric modulator for each of the above regressors we used, on each trial, ε*|Q_*CS*_*|* derived from fitting the computational model to each individual's data. These parametric modulators were based on a group-wise model fit. This allowed us to index brain activity that scaled with the strength of the observed behavioral bias in a 2 × 2 factorial design with decision (gamble or sure) and CS as main factors. We also modeled separately the following regressors of no interest: trials in which subjects chose the safe option and the CS- was displayed under the safe option, trials in which subjects chose the gamble option and the CS- was displayed under the safe option, the catch trials, and each of the pairs of the learning task that were alternated among the gamble trials. Finally, to capture residual movement-related artifacts six covariates (the three rigid-body translation and three rotations resulting from realignment) were also included as regressors of no interest.

As described, the primary aim of our neuroimaging analysis was to capture brain areas mediating the observed bias in decision making induced by a display under the safe option of either the CSwin or the CSlose predictive conditioned stimuli. The interaction contrast [(Sure/CSwin + Gamble/CSlose) − (Gamble/CSwin + Sure/CSlose)] of our parametric regressors captures this effect and allows for identification of brain areas that show high activity when subjects chose the safe option when the CSwin is displayed and when they chose the gamble option when the CSlose is displayed (see Fig. 1B). In other words our focus is on choices that account for the observed bias. Critically, a two by two interaction contrast is balanced with respect to the two main effects such that brain activity in the interaction contrast is uncontaminated by effects attributable to either the decision (safe or gamble option) or the valence associated with the CS (win or lose). Moreover, our parametric regressor weights this simple interaction by a session specific parameter that relates to the magnitude of the behavioral impact of CS's on the decision-making process. Therefore, by using a parametric regressor we test for brain areas supporting the emergence of a CS induced bias with the progression of the experiment.

Parameter estimates were used to calculate the interaction contrast for each individual subject. These contrast images were entered into a second level one-sample *t*-test across subjects (random effects analysis). Our a priori hypothesis pointed to the amygdala as the most likely candidate to mediate biases induced by presentation of CSs. First, activation of the amygdala has been found in two previous experiments studying the framing effect which is analogous to the biases that we want to explain with the present experiment (De Martino et al., 2006; Roiser et al., 2009). Moreover, a substantial animal literature has established the amygdala as important in the acquisition and expression of conditioned behaviors regardless of the valence of the CSs (Balleine and Killcross, 2006; Cardinal et al., 2002). Therefore, we restricted our primary analysis of interest to regions subsumed within an inclusive mask derived from an independent data set that found amygdala activation mediating the framing effect in subjects carrying the ss variant of the 5HTT-linked polymorphic region (Roiser et al., 2009). To build this mask we used the contrast image obtained in this previous study and thresholded it to *p* < 0.001. This mask only includes the left amygdala. If we assume that in the present experiment we are studying the same phenomenon as in Roiser et al., the laterality of the effects would be expected to be the same. Post hoc, we determined whole brain effects in our experiment, primarily for descriptive purposes, where the resulting z statistic images from the second level analysis were thresholded at *p* < 0.005 and reported in the supplementary Table S4. Significant activations are displayed by superimposition of the SPM maps on our group templates in Fig. 4 and Fig. S10.

## Results

### Cue induced biases in economic decision making

**Fig. 3:**
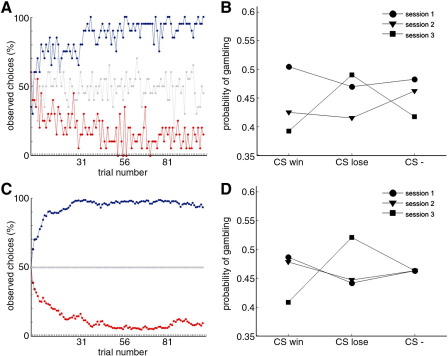
Observed and modeled results in experiment 3. (A) The observed learning curves depict, trial by trial, the proportion of subjects that chose the fractal with highest probability of monetary win (win pair) in blue, with highest probability of monetary lose (lose pair) in red, and a given fractal without any monetary outcome (neutral pair) in grey. The numbers in the x axis highlights the beginning of each session alternating gamble and learning task. (B) The probability of gambling for each of the CS displayed under the sure option in each session, shows that the preference for the gamble when the safe option was displayed over the CSwin decreased over sessions, whereas the preference for the gamble when the safe option was displayed over the CSlose increased. (C) The modeled learning curves depict, trial by trial, the probabilities of choices as simulated by a computational model based on a group-wise model fit. (D) Probability of gambling simulated by the computational model for each of the CS displayed under the sure option in each session.

During the learning task, prior to the actual gamble task, subjects had learned to choose the fractal associated with an 80% probability of winning £10 and to avoid the fractal associated with an 80% probability of losing £10 (Fig. 3A and Figs. S5A and S6A). Subjects were explicitly told that both tasks were independent and indeed, most subjects reported that the CS's displayed under the safe option did not induce a bias in their decisions (15/20 in experiment 1; 12/14 in experiment 2; and 19/20 in experiment 3).

Subjects’ choices in the gamble task were affected by the presence of the incidental conditioned fractal images, that predicted losses and wins in the separate learning task, in all three experiments (Fig. 2 and Figs. S1–S3), a finding analogous in form to what we found in previous experiments on framing (De Martino et al., 2006; Roiser et al., 2009). Specifically, in experiment 1 and 2 we found a main effect of CS (*F*(2,38) = 3.94; *P* = 0.046 l for experiment 1; and *F*(2,26) = 4.34; *P* = 0.042 for experiment 2), without any significant main effect of session (*F*(1,38) = 0.89; *p* > 0.1 for experiment 1; and *F*(2,26) = 1.59; *p* > 0.1 for experiment 2) or CS by session interaction (*F*(2,38) = 2.5; *p* > 0.1 for experiment 1; and *F*(2,26) = 1.87; *p* > 0.1 for experiment 2). Importantly, these effects had a valence directionality evident by an increased preference for the gamble option when the CS loss stimulus was presented with the sure option and a decreased preference for the gamble option when the CSwin was displayed under the sure option (For experiment 1: CSwin versus CSlose, *t*_19_ = 2.47, *p* = 0.023; CSwin versus CS-, *t*_19_ = 1.48, *p* > 0.1; CSlose versus CS-, *t*_19_ = 2.57, *p* = 0.019; for experiment 2: CSwin versus CSlose, t_13_ = 2.37, *p* = 0.034; CSwin versus CS-, t_13_ = 1.67, *p* > 0.1; CSlose versus CS-, *t*_19_ = 2.04, *p* = 0.062).

In experiment 3, although we did not observed any main effect of CS (*F*(2,38) = 0.47; *p* > 0.1) or session (*F*(2,38) = 3.2; *p* > 0.1), we observed a significant session by CS interaction (*F*(4,76) = 4.83; *P* = 0.004). This interaction reflected the emergence of the same pattern of conditioned stimulus induced preferences, that we observed in experiment 1 and 2, by the third session (CSwin versus CSlose in last session, *t*_19_ = 3.46, *p* = 0.003; CSwin versus CS- in last session, *t*_19_ = 0.9, *p* > 1; CSlose versus CS- in last session, *t*_19_ = 2.19, *p* = 0.041). Together with the emergence of the behavioral bias in session 3, we also found that subjects became gradually faster at making their decisions from session 1 to 3 as shown by a session effect on reaction time data (mean reaction time across conditions was 896.6 ms, 864.7 ms, and 810.1 ms for sessions 1, 2, and 3, respectively; *F*(2,38) = 20.2; *p* < 0.001; in the lack of CS or session by CS interaction (F2,38) = 0.5; *p* > 0.1; and *F*(4,76) = 1.6; *p* > 0.1). Although we did not find any session or session by CS interaction in experiment 1 and 2, the effects of CS were stronger in the last session for each experiment: when the effects of CS were looked separately for each session, an effect of CS on the probability of gambling was only observed in the last session of each experiment. Details in relation to how this effect evolved across sessions are detailed in Figs. S1–S3.

The source of the observed bias in the gamble task induced by the presence of the CSs cannot be accounted for by an effect on task difficulty since the presence of the different CS's did not influence subjects’ reaction times for their decisions (Experiment 1: CSwin, 1503.9 ms; CSlose, 1487.3 ms; CS-, 1496.8 ms; main effect of CS *F*(2,38) = 0.155, n.s.; Experiment 2: CSwin, 869.5 ms; CSlose, 890.9 ms; CS-, 883.8 ms; main effect of CS *F*(2,26) = 1.14, n.s.; Experiment 3: CSwin, 858.4 ms; CSlose, 860.9 ms; CS-, 852.1 ms; main effect of CS *F*(2,38) = 0.5; n.s.). Moreover, subjects’ attention to the task persisted throughout the entire experiment evident in the fact that they made accurate choices according to the expected value of the different options in catch trials (Fig. S4).

### Amygdala activation and expression of behavioral biases

**Fig. 4:**
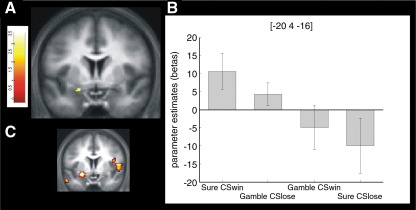
fMRI results. (A) Interaction of parametric regressors [(Sure/CSwin + Gamble/CSlose) − (Gamble/CSwin + Sure/CSlose)]; brain activation reflecting the emergence of the CS induced bias with the progression of the experiment, namely choosing the gamble option when the CS loss is presented and choosing the safe option when CSwin is presented over the safe option and weighted by a session specific parameter that relates to the magnitude of the behavioral impact of CS's on the decision making process. This analysis was constrained by an inclusive mask using a similar interaction derived from an independent data set that found amygdala activation for behavioral biases in decision making (see text for details). A cluster of highly significant activation was observed within this mask in the left amygdala (peak Z score = 3.27; *P* = 0.015 FWE; *P* = 0.001 uncorrected). (B) Parameter estimates at the peak coordinates of the cluster. Coordinates are given in MNI space. Error bars indicate SEM. (C) Display of the used inclusive mask at the same y coordinates as A and B.

We found left amygdala [Montreal Neurological Institute (MNI) space coordinates (x,y,z) −20,4,−16; peak Z score = 3.27; *p* = 0.001 uncorrected; *p* = 0.015 *FWE*] (Fig. 4) expressed a significant interaction between decision (gamble or not) and the parameter ε*|Q_*CS*_*|*, which indicated the magnitude of the effect of the CS on the decision process. The left amygdala was activated in conditions where the presence of the CSlose under the sure option biased subjects to choose the gamble option and where the CSwin under the sure option biased subjects away from the gamble option. Although the mask that we used only included the left amygdala, we did not observe any activation in the right amygdala when performing an exploratory analysis at a more liberal threshold, suggesting that in the present experiment the amygdala response showed a left-sided laterality, as found previously in an fMRI study of the framing effect (Roiser et al., 2009). For descriptive purposes, we also determined post hoc whole brain effects of this interaction contrast (*p* < 0.005 uncorrected, supplementary Table S4). Remarkably, we also found a large cluster of activation in the right ventral striatum (anterior caudate) [MNI space coordinates12, 22, 0; peak Z score = 3.34; *p* < 0.001, uncorrected].

## Discussion

We show that the incidental presentation of stimuli that predict wins and losses can bias independent economic decision making under uncertainty. These influences of conditioned stimuli we observe is similar to an influence elicited by mere presentation of gain and lose semantic frames as seen in classical framing experiments. Moreover, the emergence of this bias was related to amygdala activation which shows that the striking similarity between the behavioral effects, observed with the present paradigm and classical framing effects, also extends to its neurobiological underpinnings.

It is known that reward and punishment conditioned stimuli generate approach and withdrawal responses towards the stimuli (see Everitt et al., 2003; Kim and Jung, 2006) for reviews in appetitive and aversive conditioning, respectively). The increased preference towards the gamble option when the CSlose was presented under the sure option can be conceived of as a withdrawal from a stimulus that predicts a loss. On the other hand, the decreased preference towards the gamble option observed when the CSwin was presented under the sure option can be conceived of as enhanced approach to a stimulus that has been learned to predict wins. Therefore, we suggest that after learning an association between stimuli and outcome through instrumental conditioning in the learning task, the CSwin and the CSlose generate expectations of reward and punishment. These automatically access a Pavlovian system, which triggers an associated unconditioned response such as approach or withdrawal which we suggest is the mechanism by which the CSs induce the observed decision biases in the unrelated gamble task.

Note that although our training task was instrumental it is widely accepted that the type of conditioning we implemented involves both Pavlovian and instrumental mechanisms (Kim and Jung, 2006; Mackintosh, 1983). We are aware that it is difficult to disentangle what is purely Pavlovian or instrumental in our experimental setting. However, in the critical test phase it was the mere presence of these predictive stimuli, without a requirement to perform a learnt action that was critical to the decision biasing effect we observed. This behavioral effect is then reminiscent of a Pavlovian instrumental transfer (PIT) experiments in which the amount of responses in an instrumental task to obtain reward is increased by the mere presentation of a conditioned stimulus that has been learned to predict reward (Lovibond, 1983) and the instrumental response to avoid punishment is increased by the mere presentation of a conditioned stimulus that has been learned to predict punishment (Overmier et al., 1971).

The involvement of the amygdala in generating decision biases points to a key role for the Pavlovian system, a system also implicated in sign tracking (Parkinson et al., 2000), fear conditioning (Phelps and LeDoux, 2005), the generation of Pavlovian to instrumental interactions including conditioned reinforcement (Burns et al., 1993) and an invigorating effect on instrumental actions (Hall et al., 2001; Talmi et al., 2008). The overarching role of the Pavlovian system in these diverse contexts can be conceptualised in terms of information provision in relation to motivational state to an otherwise motivationally less flexible habitual system (Dayan and Balleine, 2002). We acknowledge that other brain areas may also be implicated in the observed effects. However, our a priori hypothesis was restricted to the amygdala and as shown by our exploratory analysis the caudate and the insula were also activated in the expression of the decision bias driven by the conditioned fractal images. Indeed, the striatum is a key brain area implicated in the expression of Pavlovian/instrumental interactions in animals (Cardinal et al., 2002) and humans (Talmi et al., 2008). It has been suggested that the neural mechanism that generates Pavlovian/instrumental interactions requires translation of an affective signal, mediated by amygdala, to processes implemented in the striatum. One potential mechanism by which this could be achieved is through amygdala projections to the dopaminergic midbrain that would then modulate neuronal processing in the striatum (Balleine and Killcross, 2006; Cardinal et al., 2002).

It is of interest that the impact of conditioned stimuli in experiment 3 became stronger as the experiment progressed. As subjects become more experienced with the gamble task they are likely to rely more on habitual or automated mechanisms in making their decisions, a context where the influences of conditioned associations on instrumental performance is known to be more robust (Dickinson and Balleine, 2001). As the habitual system does not use an explicit model of the outcomes of actions, the Pavlovian and instrumental representations of value interact more closely when a behavior becomes habitual, resulting in larger PIT effects when subjects rely more on habitual mechanisms to make decisions in the gamble task. A general view on habit formation assumes that behavior begins as goal directed but becomes habitual through reinforcement in a stable environment. For example, after habituation has taken place, the behavior is no longer influenced by outcome devaluation, a hallmark of goal directedness (Dickinson & Balleine, 2001). Although subjects were never reinforced in the gamble task, the latency effect, where subjects became gradually faster at making decisions from session 1 to 3, is consistent with findings from skill learning that performing an action many times, even without receiving explicit reinforcement, leads to this action becoming automated, as expressed in habit (Doyon et al., 2003). Therefore, our data supports the suggestion that subjects’ decision process became more automated as the task progressed and thereby become more susceptible to conditioned influences. In experiment 1 and 2 the behavioral bias elicited by the CS was much stronger and observed over all sessions without any apparent interaction with session. In the context of scanning this behavioral effect showed an interaction with session, and was only significant at a group level during the third session. However, we also observed in both our preliminary behavioral studies (experiments 1 and 2) that the effect of the CS on decision making became stronger as the experiment progressed. Whereas experiment 1 and 2 were performed inside normal experimental rooms, experiment 3 was carried out inside the scanner and this change in environment may well explain the differences in the emergent pattern of behavior seen across the three different experiments. For example, the novelty and ambient noise of the scanner environment may have delayed the emergence of a more habit based approach to performing the gamble task.

Our data provides convergent behavioral and neurobiological evidence that a bias in decision making reflects the dominance of a Pavlovian valuation system on goal-directed decision making under uncertainty. Incidental stimuli that predict losses and wins in an entirely different context to that of the gamble task, appear to have generated avoidance and approach tendencies with respect to the sure option and, in so doing, distorted subjects’ behavioural preferences. These distortions result in a context bias identical in form to that reported in the framing effect (De Martino et al., 2006; Roiser et al., 2009). The fact that framing tasks use emotionally laden words, such as ‘lose’ and ‘win,’ semantics known to generate approach and avoidance tendencies (Chen and Bargh, 1999), supports our suggestion that decision biases can arise solely out of a conditioned effect. Although our computational model also supports this view, the modelling results need to be taken with caution because alternative models have not been tested and the model fit to the data is often suboptimal. In terms of mechanisms we suggest that Pavlovian action tendencies approach or avoid, generated by stimuli that predict wins or losses bias the level of control exercised by an instrumental system that, rationally, should not care about these irrelevant stimuli. Emotional laden words whose meaning has been experienced repeatedly over the entire lifespan may act as much stronger conditioned association than our fractal images that were newly associated with their respective outcomes. This could potentially explain why the standard framing effect as studied with manipulations of the wording of the different choice options is much stronger than the effects that we observed and can be seen after a single presentation (Kahneman et al., 1991; Tversky and Kahneman, 1981, 1986). Although in the present work we show how the Pavlovian system can be a source of distortion in economic decision making, it is worth noting that these kind of biases are likely to be of evolutionary benefit in so far as they are conserved across phylogeny where they are likely to provide, on average, reliable predictions about expected reward and punishments in situations of uncertainty.

## Appendix A Supplementary data

Fig. S1 Behavioral results for experiment 1. Each bar represents, for each individual subject, the difference between the probabilities of choosing the gamble in two different CS conditions in each session. A value of zero represents a complete indifference for the CS manipulation. Positive numbers denote the existence of the hypothesized behavioral bias whereas negative numbers denote an opposite effect. In this figure it can be observed that the hypothesized bias is stronger in the second part of the experiment. Note that the main effect of CS was only significant in session 2 (main effect of CS in session 1: *F*(2,38) = 1.39, *p* > 0.1; main effect of CS in session 2: *F*(2,38) = 5.13, *p* = 0.026).

Fig. S2 Behavioral results for experiment 2. Each bar represents for each individual subject the difference between the probabilities of choosing the gamble in two different CS conditions in each session. A value of zero represents a complete indifference for the CS manipulation. Positive numbers denote the existence of the hypothesized behavioral bias whereas negative numbers denote an opposite effect. In this figure it can be observed that the hypothesized bias is stronger as the experiment progresses. Note that the main effect of CS was only significant in session 3 (main effect of CS in session 1: *F*(2,26) = 1.56, *p* > 0.1; main effect of CS in session 2: *F*(2,26) = 1.47, *p* > 0.1; main effect of CS in session 3: *F*(2,26) = 5.71, *p* = 0.023).

Fig. S3 Behavioral results for experiment 3 (inside the scanner). Each bar represents for each individual subject the difference between the probabilities of choosing the gamble in two different CS conditions in each session. A value of zero represents a complete indifference for the CS manipulation. Positive numbers denote the existence of the hypothesized behavioral bias whereas negative numbers denote an opposite effect. In this figure it can be observed that the hypothesized bias is stronger as the experiment progresses. Note that the main effect of CS was only significant in session 3 (main effect of CS in session 1: *F*(2,38) = 0.81, *p* > 0.1; main effect of CS in session 2: *F*(2,38) = 1.69, *p* > 0.1; main effect of CS in session 3: *F*(2,38) = 5.78, *p* = 0.006).

Fig. S4 Behavioral results for catch trials in all experiments. In these trials (25% of all gamble trials) the expected outcome of the safe and gamble option was markedly unbalanced. In half of these trials (depicted in yellow) the safe option had higher expected outcome as the probability of winning the gamble was only 0.05. In the other half of these trials (depicted in blue) the gamble option had higher expected outcome as the probability of winning the gamble was 0.95. The plots show the probability of choosing the gamble in these trials for each of the CS depicted under the safe option. The subjects were highly accurate in making optimal choices in these trials, providing evidence of continued engagement with the task throughout the experiment.

Fig. S5 Observed results of the learning and the three sessions of the gamble task in the experiment 1 and comparison to the modeled behavioral choices. (**A**) The learning curves depict, trial by trial, the proportion of subjects that chose the fractal with highest probability of monetary win (win pair) in blue, the proportion of subjects that chose the fractal with highest probability of monetary lose (lose pair) in red, and the proportion of subjects that chose a given fractal without any monetary outcome (neutral pair) in grey. The numbers in the x axis highlights the beginning of each session. (**B**) The probability of gambling for each of the CS displayed under the sure option in each session, shows that the preference for the gamble when the safe option was displayed over the CSlose increased in the second session, whereas the preference for the gamble when the safe option was displayed over the CSwin slightly decreased. There is a main effect of CS (*F*(2,38) = 3.94; *P* = 0.046l) and the session by CS interaction only shows a trend: *F*(2,38) = 2.5; *P* = 0.095). (**C**) The modeled learning curves depict, trial by trial, the probabilities of choices simulated by the computational model based on a group-wise model fit. (**D**) Probability of gambling simulated by the computational model for each of the CS displayed under the sure option in each session.

Fig. S6 Observed results of the learning and the three sessions of the gamble task in the experiment 2 and comparison to the modeled behavioral choices. (**A**) The learning curves depict, trial by trial, the proportion of subjects that chose the fractal with highest probability of monetary win (win pair) in blue, the proportion of subjects that chose the fractal with highest probability of monetary lose (lose pair) in red, and the proportion of subjects that chose a given fractal without any monetary outcome (neutral pair) in grey. The numbers in the x axis highlights the beginning of each session. (**B**) The probability of gambling for each of the CS displayed under the sure option in each session, shows that the preference for the gamble when the safe option was displayed over the CSwin decreased over sessions, whereas the preference for the gamble when the safe option was displayed over the CSlose did not change. In this experiment there was a main effect of CS (*F*(2,26) = 4.34; *P* = 0.042), and the session by CS interaction was not significant: *F*(4,52) = 1.87; *P* = 0.131). (**C**) The modeled learning curves depict, trial by trial, the probabilities of choices simulated by the computational model based on a group-wise model fit. (**D**) Probability of gambling simulated by the computational model for each of the CS displayed under the sure option in each session.

Fig. S7 Individual subjects parameters for experiment 1. The bar represents the mean value for each parameter, the error bar represents the standard deviation, and the dots represent the individual subjects’ parameters. (**A**) Alfa parameter (learning rate) for the learning task. Note that this parameter was fitted separately for the learning of the CSwin (reward) and the CSlose (punishment). (**B**) Beta parameter (temperature) for the learning task. Note that this parameter was fitted separately for the learning of the CSwin (reward) and the CSlose (punishment). (**C**) Epsilon parameter (bias strength) for the gamble task. Note that this parameter was allowed to change between sessions.

Fig. S8 Individual subjects parameters for experiment 2. The bar represents the mean value for each parameter, the error bar represents the standard deviation, and the dots represent the individual subjects’ parameters. (**A**) Alfa parameter (learning rate) for the learning task. Note that this parameter was fitted separately for the learning of the CSwin (reward) and the CSlose (punishment). (**B**) Beta parameter (temperature) for the learning task. Note that this parameter was fitted separately for the learning of the CSwin (reward) and the CSlose (punishment). (**C**) Epsilon parameter (bias strength) for the gamble task. Note that this parameter was allowed to change between sessions.

Fig. S9 Individual subjects parameters for experiment 3. The bar represents the mean value for each parameter, the error bar represents the standard deviation, and the dots represent the individual subjects’ parameters. (**A**) Alfa parameter (learning rate) for the learning task. Note that this parameter was fitted separately for the learning of the CSwin (reward) and the CSlose (punishment). (**B**) Beta parameter (temperature) for the learning task. Note that this parameter was fitted separately for the learning of the CSwin (reward) and the CSlose (punishment). (**C**) Epsilon parameter (bias strength) for the gamble task. Note that this parameter was allowed to change between sessions and only differed from 0 for the last session in line with the descriptive emergence of the key behavioral bias (mean ε in session 1 was − 0.01, *t*_19_ = 0.7, *p* > 0.1; mean ε in session 2 was −0.007, *t*_19_ = 0.75, *p* > 0.1; mean ε in session 3 was 0.03, *t*_19_ = 2.9, *p* = 0.009).

Fig. S10 fMRI results. Interaction [(Sure/CSwin + Gamble/CSlose) − (Gamble/CSwin + Sure/CSlose)]; brain activation reflecting the observed bias in subjects’ choices, that is choosing the gamble option when the CSlose is presented under the safe option and choosing the safe option when CSwin is presented under the safe option. Clusters of activation are observed in the right caudate [(x,y,z) = 12,22,0; peak Z score = 3.34; *p* < 0.001 uncorrected] and right insula [(x,y,z) = 50,12,−8; peak Z score = 3.02; *p* < 0.001 uncorrected]. The statistical parametric maps are thresholded at *p* < 0.005.

Table S1 Quality of behavioral fits for the computational models of the learning and the gamble task in experiment 1. Pseudo-*R*^2^ is defined as (*r* − *l*)/*r* where *l* and *r* are, respectively, the log likelihoods of the data under the model and under purely random choices (0.5 for every trial).

Table S2 Quality of behavioral fits for the computational models of the learning and the gamble task in experiment 2. Pseudo-*R*^2^ is defined as (*r* - *l*)/*r* where *l* and *r* are, respectively, the log likelihoods of the data under the model and under purely random choices (0.5 for every trial).

Table S3 Quality of behavioral fits for the computational models of the learning and the gamble task in experiment 2. Pseudo-*R*^2^ is defined as (*r* − *l*)/*r* where *l* and *r* are, respectively, the log likelihoods of the data under the model and under purely random choices (0.5 for every trial).

Table S4 Brain areas active in the interaction contrast [(Sure/CSwin + Gamble/CSlose) − (Gamble/CSwin + Sure/CSlose)]. Data is thresholded at *p* < 0.001 uncorrected for multiple comparisons.
