# Supplementary material for: Conditioned associations and economic decision biases
Source: Neuroimage. 2010 Oct 15;53(1-2):206–14. doi: 10.1016/j.neuroimage.2010.06.021 (PMC2923756; doi:10.1016/j.neuroimage.2010.06.021)
Supplement: Fig. S1 — Behavioral results for experiment 1. Each bar represents, for each individual subject, the difference between the probabilities of choosing the gamble in two different CS conditions in each session. A value of zero represents a complete indifference for the CS manipulation. Positive numbers denote the existence of the hypothesized behavioral bias whereas negative numbers denote an opposite effect. In this figure it can be observed that the hypothesized bias is stronger in the second part of the experiment. Note that the main effect of CS was only significant in session 2 (main effect of CS in session 1: F(2,38) = 1.39, p > 0.1; main effect of CS in session 2: F(2,38) = 5.13, p = 0.026). [file mmc1.doc]

**SUPPLEMENTAL DATA**

**Figure S1**: Behavioral results for experiment 1. Each bar represents, for each individual subject, the difference between the probabilities of choosing the gamble in two different CS conditions in each session. A value of zero represents a complete indifference for the CS manipulation. Positive numbers denote the existence of the hypothesized behavioral bias whereas negative numbers denote an opposite effect. In this figure it can be observed that the hypothesized bias is stronger in the second part of the experiment. Note that the main effect of CS was only significant in session 2 (main effect of CS in session 1: F(2,38)=1.39, p>0.1; main effect of CS in session 2: F(2,38)=5.13, p=0.026).


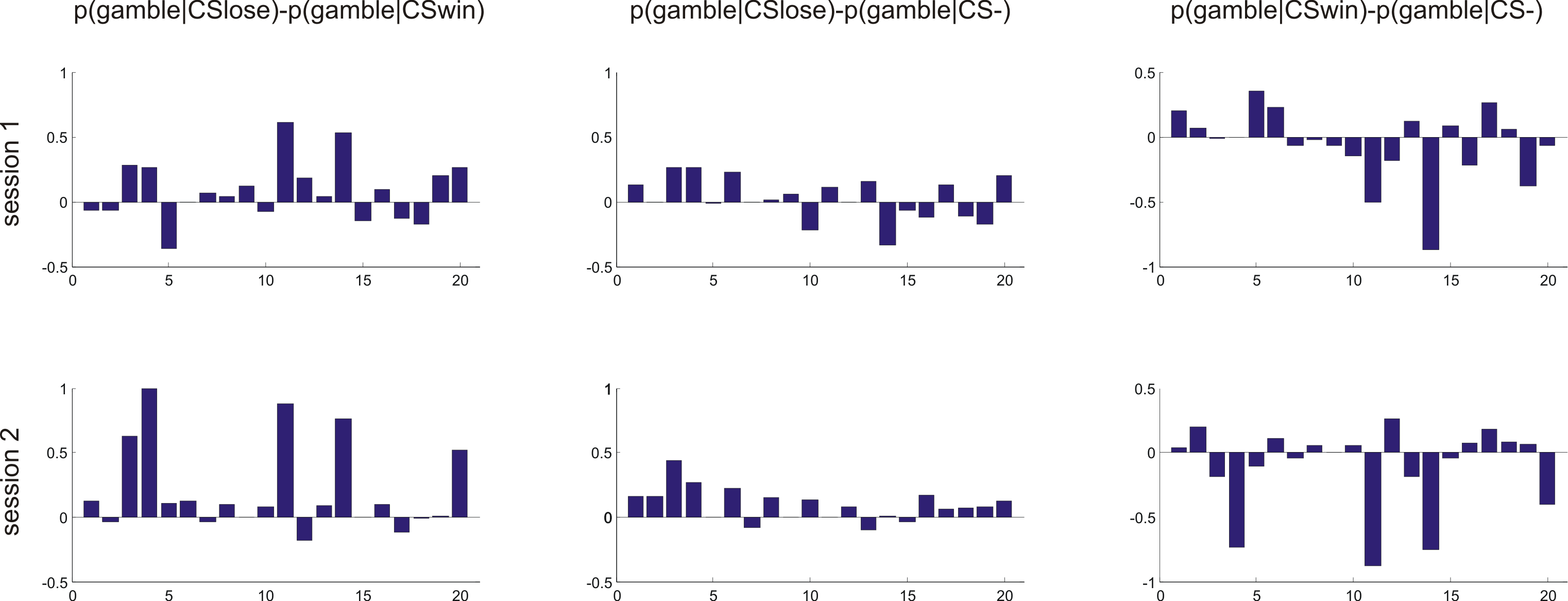


**Figure S2**: Behavioral results for experiment 2. Each bar represents for each individual subject the difference between the probabilities of choosing the gamble in two different CS conditions in each session. A value of zero represents a complete indifference for the CS manipulation. Positive numbers denote the existence of the hypothesized behavioral bias whereas negative numbers denote an opposite effect. In this figure it can be observed that the hypothesized bias is stronger as the experiment progresses. Note that the main effect of CS was only significant in session 3 (main effect of CS in session 1: F(2,26)=1.56, p>0.1; main effect of CS in session 2: F(2,26)=1.47, p>0.1; main effect of CS in session 3: F(2,26)=5.71, p=0.023).

**
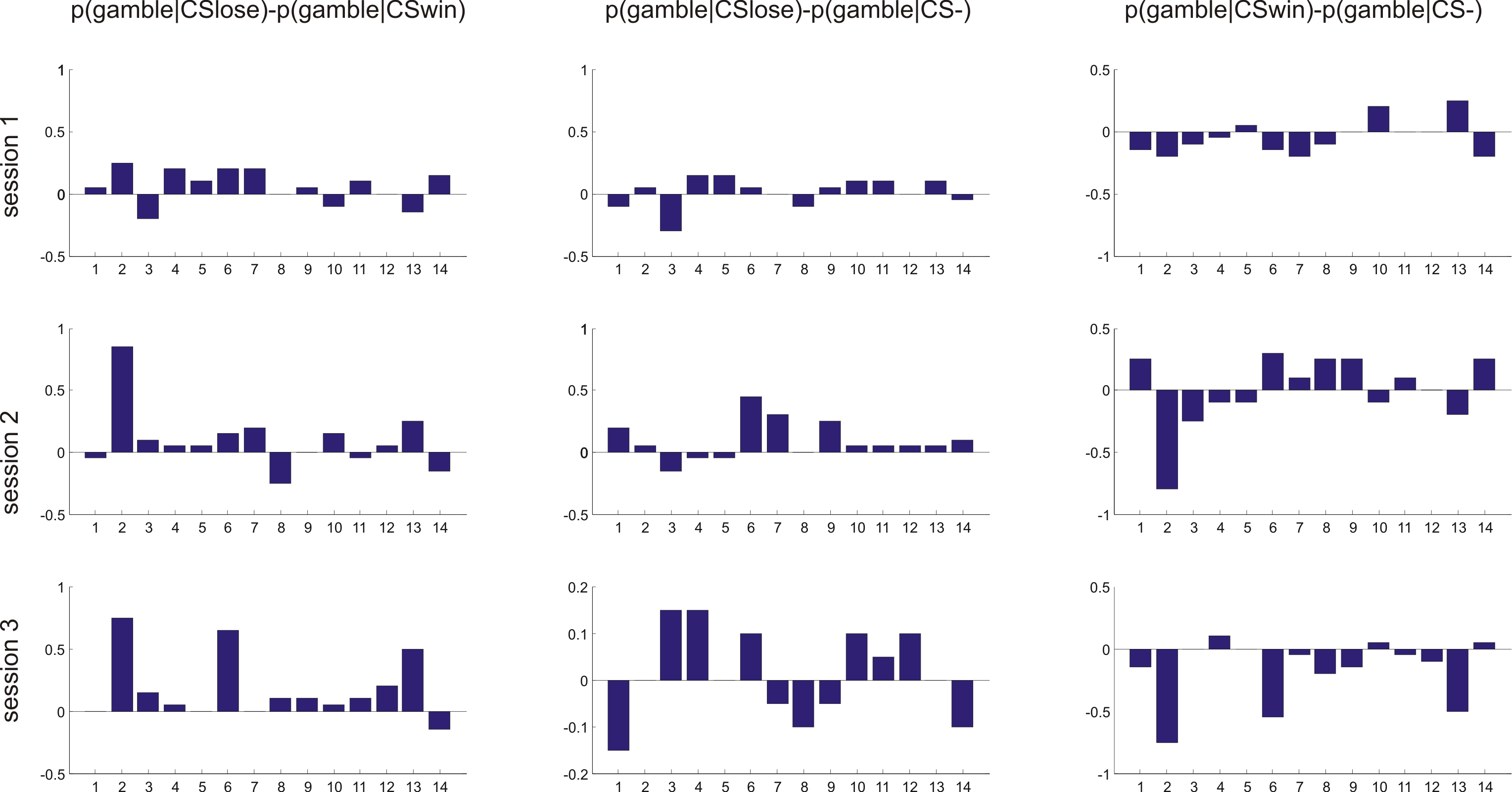
**

**Figure S3**: Behavioral results for experiment 3 (inside the scanner). Each bar represents for each individual subject the difference between the probabilities of choosing the gamble in two different CS conditions in each session. A value of zero represents a complete indifference for the CS manipulation. Positive numbers denote the existence of the hypothesized behavioral bias whereas negative numbers denote an opposite effect. In this figure it can be observed that the hypothesized bias is stronger as the experiment progresses. Note that the main effect of CS was only significant in session 3 (main effect of CS in session 1: F(2,38)=0.81, p>0.1; main effect of CS in session 2: F(2,38)=1.69, p>0.1; main effect of CS in session 3: F(2,38)=5.78, p=0.006).

**
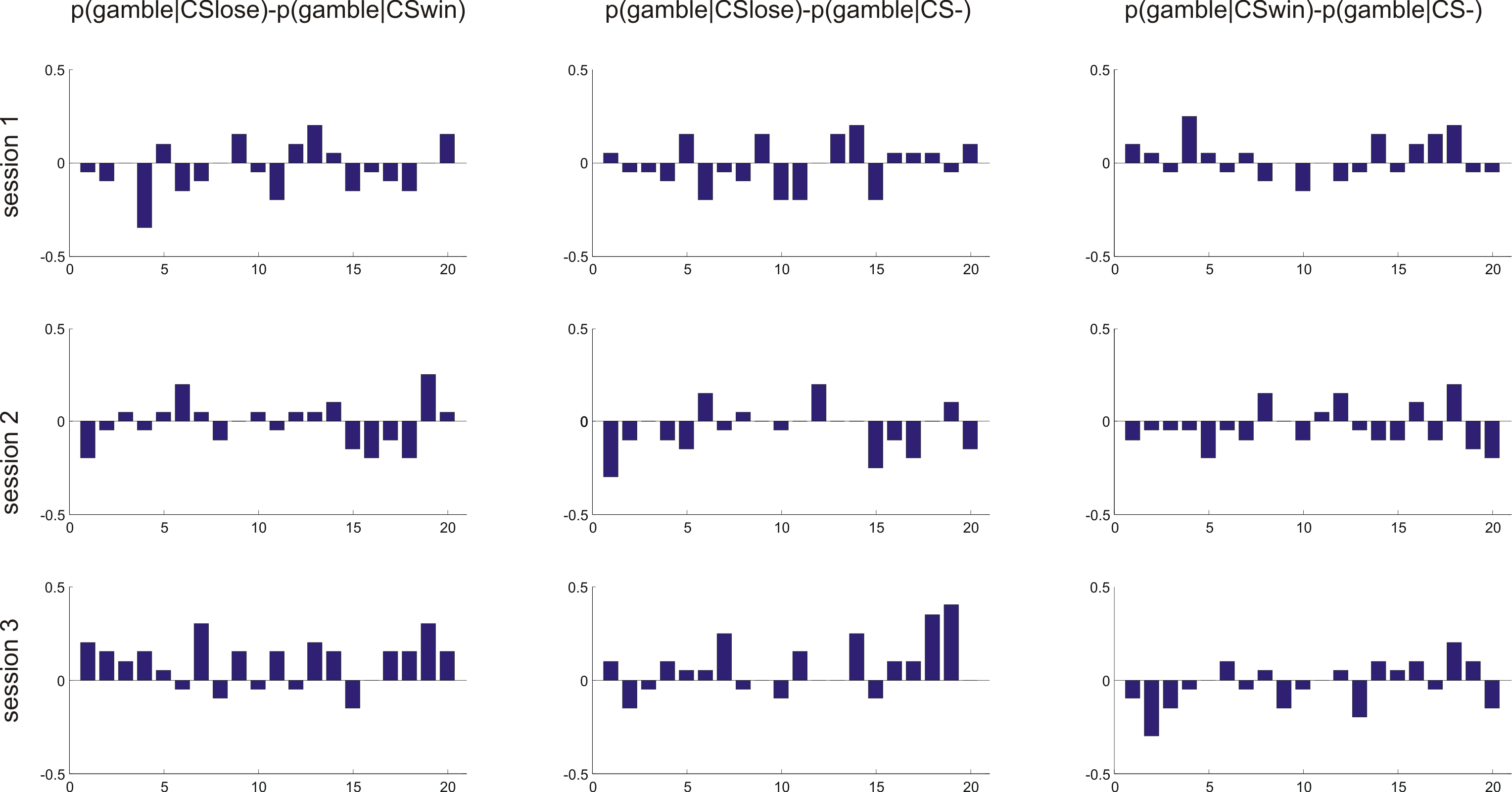
**

**Figure S4**: Behavioral results for catch trials in all experiments. In these trials (25% of all gamble trials) the expected outcome of the safe and gamble option were markedly unbalanced. In half of these trials (depicted in yellow) the safe option had higher expected outcome as the probability of winning the gamble was only 0.05. In the other half of these trials (depicted in blue) the gamble option had higher expected outcome as the probability of winning the gamble was 0.95. The plots show the probability of choosing the gamble in these trials for each of the CS depicted under the safe option. The subjects were highly accurate in making optimal choices in these trials, providing evidence of continued engagement with the task throughout the experiment.


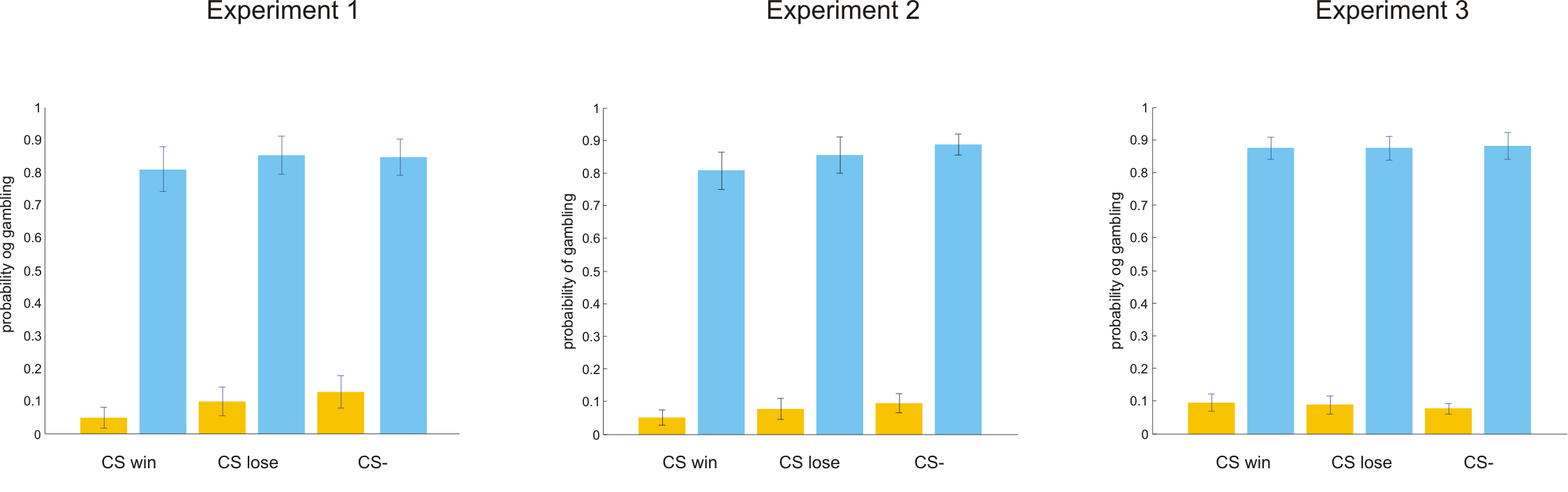


**Figure S5**: Observed results of the learning and the three sessions of the gamble task in the experiment 1 and comparison to the modeled behavioral choices.

(A) The learning curves depict, trial by trial, the proportion of subjects that chose the fractal with highest probability of monetary win (win pair) in blue, the proportion of subjects that chose the fractal with highest probability of monetary lose (lose pair) in red, and the proportion of subjects that chose a given fractal without any monetary outcome (neutral pair) in grey. The numbers in the x axis highlights the beginning of each session. (**B**) The probability of gambling for each of the CS displayed under the sure option in each session, shows that the preference for the gamble when the safe option was displayed over the CS-lose increased in the second session, whereas the preference for the gamble when the safe option was displayed over the CS-win slightly decreased. There is a main effect of CS (F(2,38)=3.94; P=0.046l) and the session by CS interaction only shows a trend: F(2,38)=2.5; P=0.095).

(**C**) The modeled learning curves depict, trial by trial, the probabilities of choices simulated by the computational model *based on a group-wise model fit*.

(**D**) Probability of gambling simulated by the computational model for each of the CS displayed under the sure option in each session.


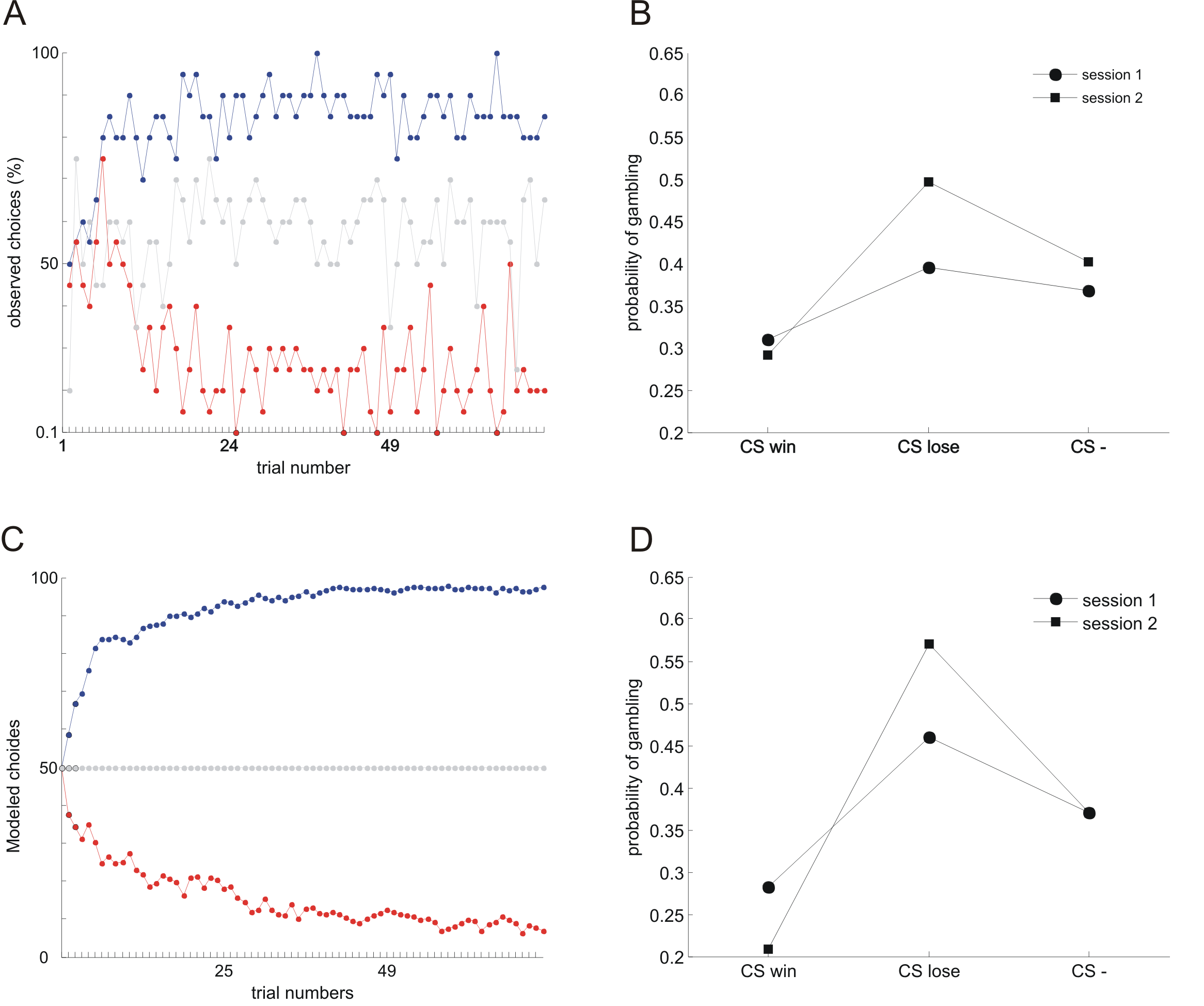


**Figure S6**: Observed results of the learning and the three sessions of the gamble task in the experiment 2 and comparison to the modeled behavioral choices.

(**A**) The learning curves depict, trial by trial, the proportion of subjects that chose the fractal with highest probability of monetary win (win pair) in blue, the proportion of subjects that chose the fractal with highest probability of monetary lose (lose pair) in red, and the proportion of subjects that chose a given fractal without any monetary outcome (neutral pair) in grey. The numbers in the x axis highlights the beginning of each session.

(**B**) The probability of gambling for each of the CS displayed under the sure option in each session, shows that the preference for the gamble when the safe option was displayed over the CS-win decreased over sessions, whereas the preference for the gamble when the safe option was displayed over the CS-lose did not change. In this experiment there was a main effect of CS (F(2,26)=4.34; P=0.042), and the session by CS interaction was not significant: F(4,52)=1.87; P=0.131).

(**C**) The modeled learning curves depict, trial by trial, the probabilities of choices simulated by the computational model *based on a group-wise model fit*.

(**D**) Probability of gambling simulated by the computational model for each of the CS displayed under the sure option in each session.


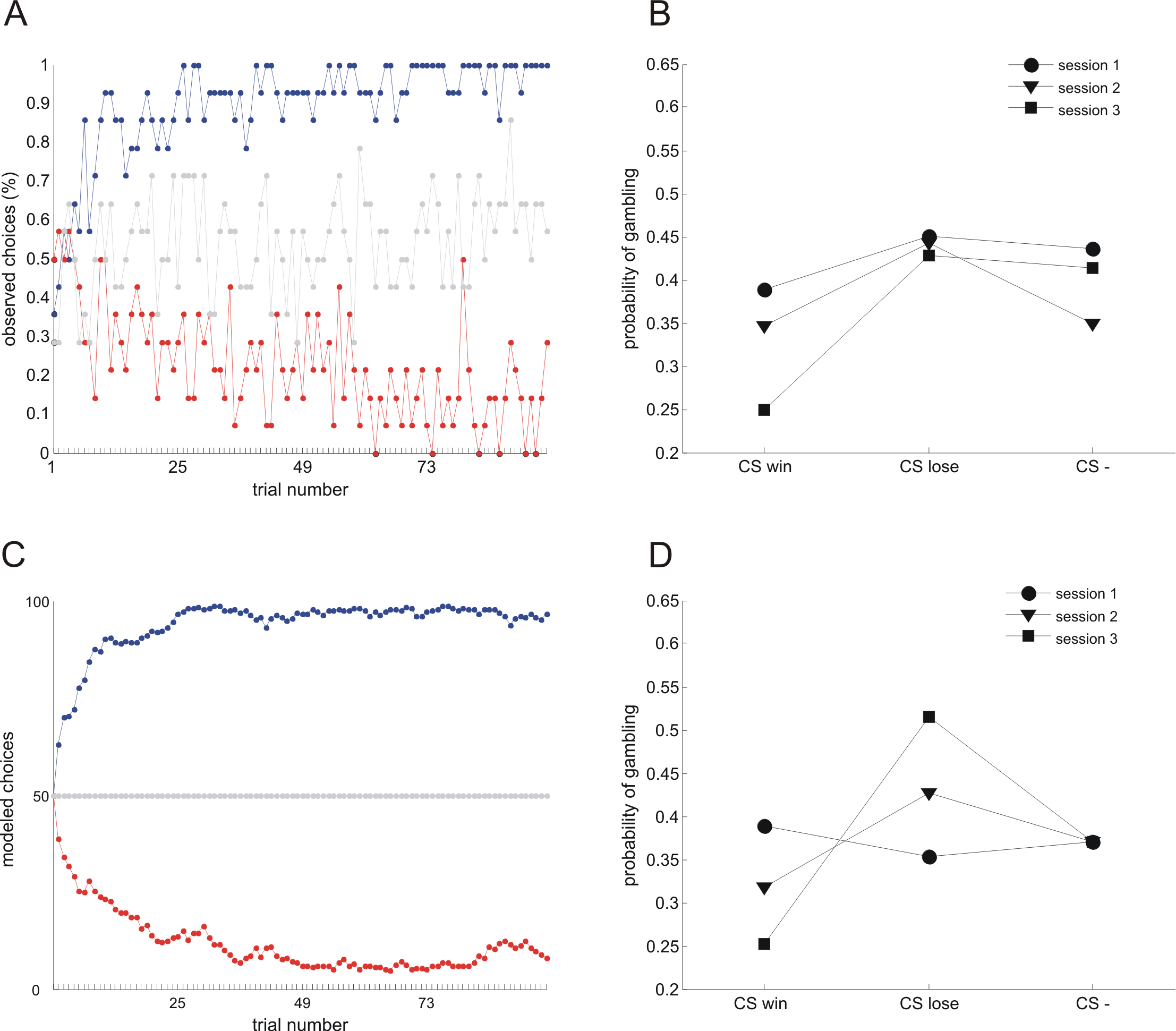


***Figure S7****: Individual subjects parameters for experiment 1. The bar represents the mean value for each parameter, the error bar represents the standard deviation, and the dots represent the individual subjects’ parameters.*

*(A) Alfa parameter (learning rate) for the learning task. Note that this parameter was fitted separately for the learning of the CS win (reward) and the CS lose (punishment).*

*(B) Beta parameter (temperature) for the learning task. Note that this parameter was fitted separately for the learning of the CS win (reward) and the CS lose (punishment).*

*(C) Epsilon parameter (bias strength) for the gamble task. Note that this parameter was allowed to change between sessions.*


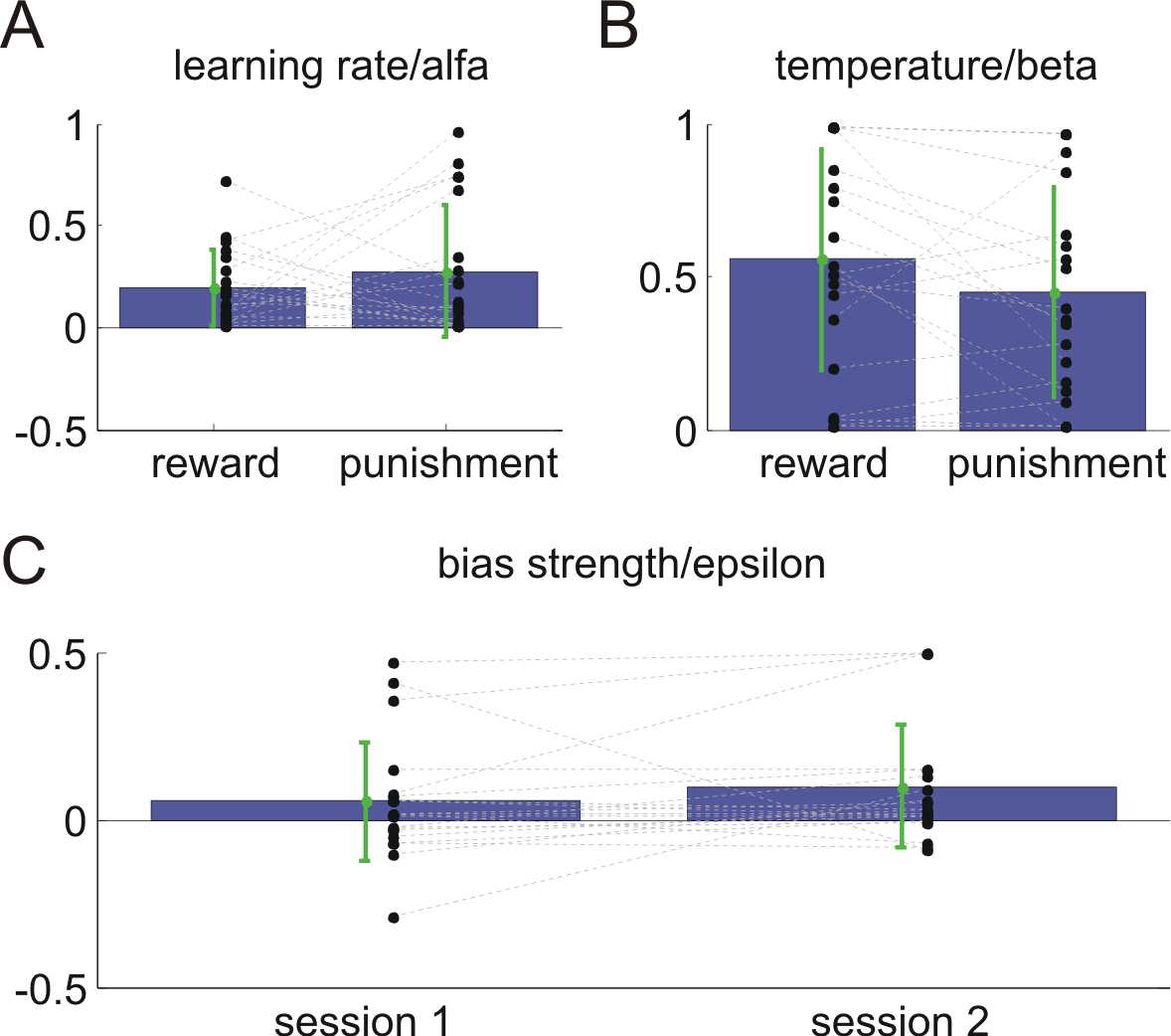


***Figure S8****: Individual subjects parameters for experiment 2. The bar represents the mean value for each parameter, the error bar represents the standard deviation, and the dots represent the individual subjects’ parameters.*

*(A) Alfa parameter (learning rate) for the learning task. Note that this parameter was fitted separately for the learning of the CS win (reward) and the CS lose (punishment).*

*(B) Beta parameter (temperature) for the learning task. Note that this parameter was fitted separately for the learning of the CS win (reward) and the CS lose (punishment).*

*(C) Epsilon parameter (bias strength) for the gamble task. Note that this parameter was allowed to change between sessions.*


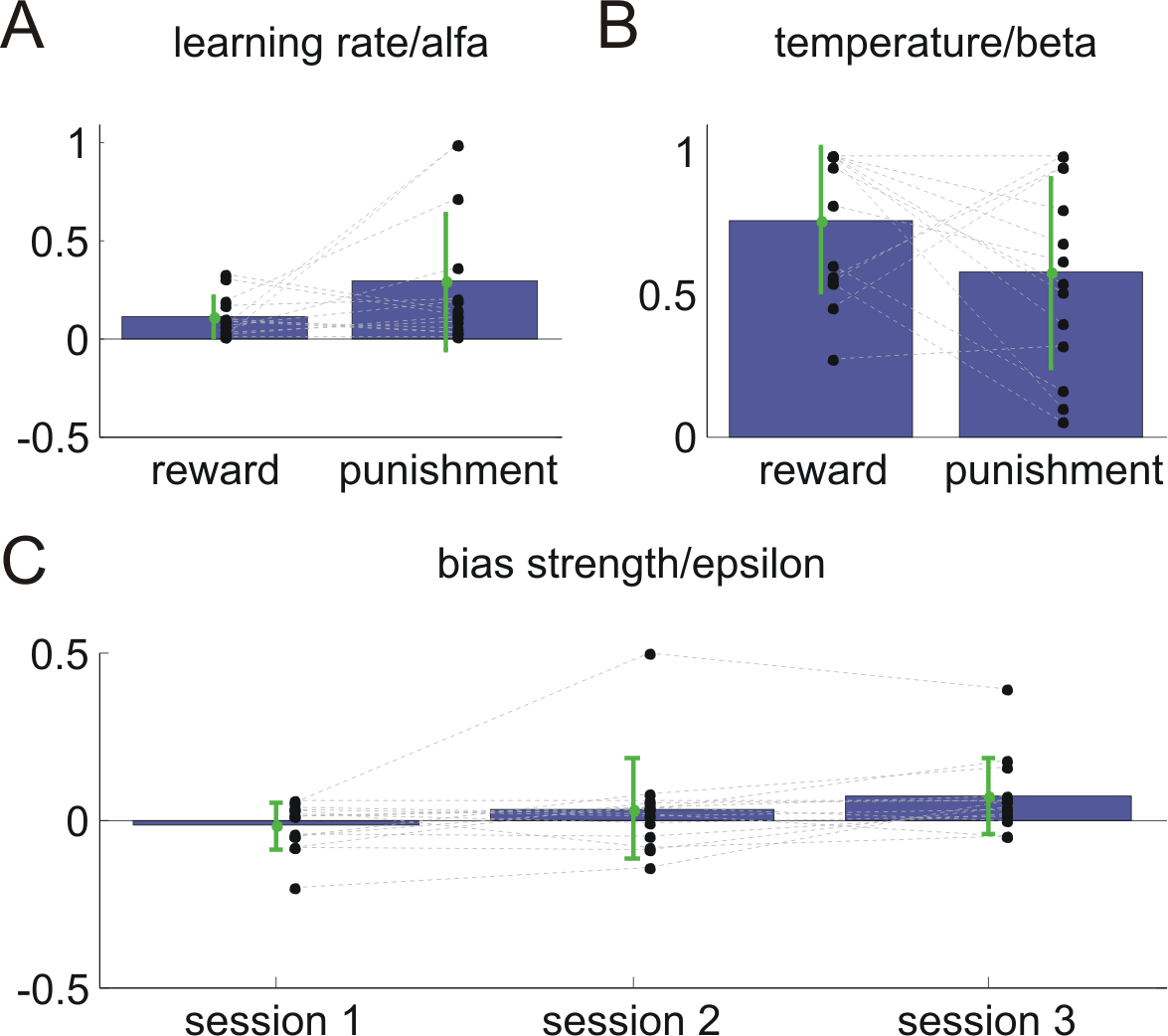


***Figure S9****: Individual subjects parameters for experiment 3. The bar represents the mean value for each parameter, the error bar represents the standard deviation, and the dots represent the individual subjects’ parameters.*

*(A) Alfa parameter (learning rate) for the learning task. Note that this parameter was fitted separately for the learning of the CS win (reward) and the CS lose (punishment).*

*(B) Beta parameter (temperature) for the learning task. Note that this parameter was fitted separately for the learning of the CS win (reward) and the CS lose (punishment).*

*(C) Epsilon parameter (bias strength) for the gamble task. Note that this parameter was allowed to change between sessions and only differed from 0 for the last session in line with the descriptive emergence of the key behavioral bias (mean ε in session 1 was -0.01, t_19_=0.7, p>0.1; mean ε in session 2 was -0.007, t_19_=0.75, p>0.1; mean ε in session 3 was 0.03, t_19_=2.9, p=0.009).*


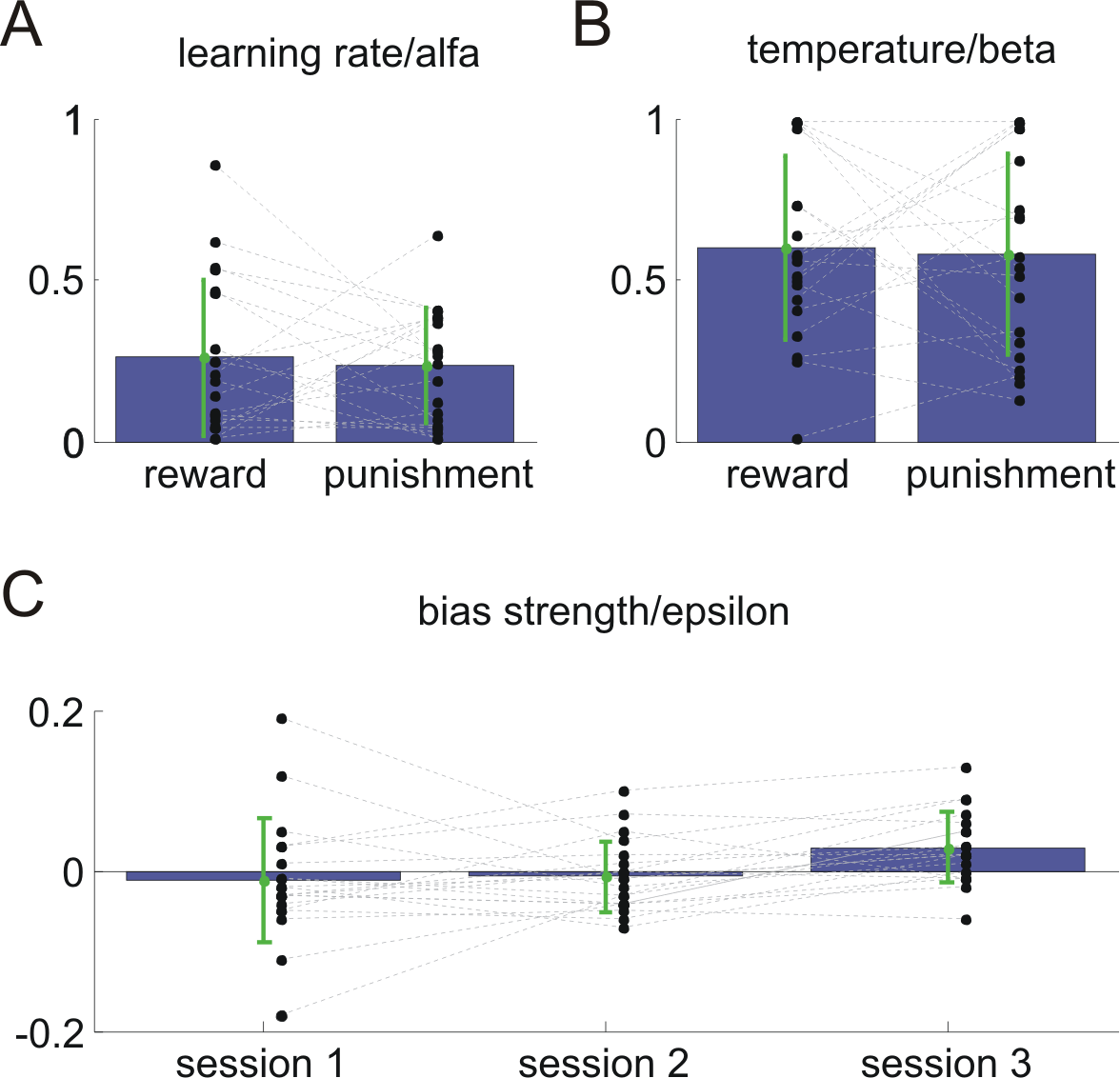


**Figure S10**: fMRI results. Interaction [(Sure/CSwin + Gamble/CSlose) – (Gamble/CSwin + Sure/CSlose)]; brain activation reflecting the observed bias in subjects’ choices, that is choosing the gamble option when the CSlose is presented under the safe option and choosing the safe option when CSwin is presented under the safe option. Clusters of activation are observed in the right caudate [(x,y,z)=12,22,0; peak Z score=3.34; p<0.001 uncorrected] and right insula [(x,y,z)=50,12,-8; peak Z score=3.02; p<0.001 uncorrected]. The statistical parametric maps are thresholded at p<0.005.

**
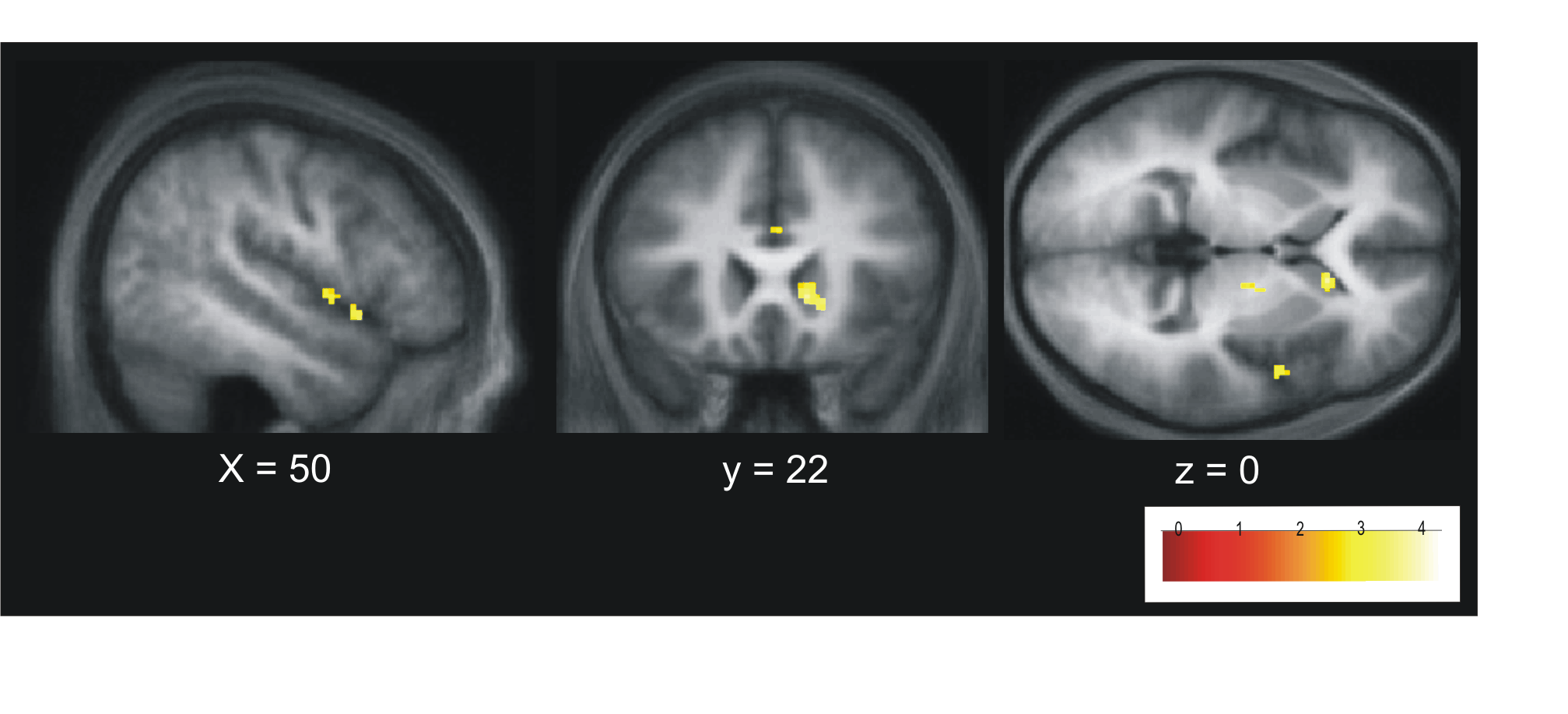
**

**Supplemental Tables**

***Table S1:*** *Quality of behavioral fits for the computational models of the learning and the gamble task in experiment 1. Pseudo-R^2^ is defined as (r-l)/r where l and r are, respectively, the log likelihoods of the data under the model and under purely random choices (0.5 for every trial).*

| subject | Learning task: pseudo R^2^ | Gamble task: pseudo R^2^ |
| --- | --- | --- |
| 1 | 0.00 | 0.12 |
| 2 | -0.08 | -0.11 |
| 3 | 0.95 | 0.07 |
| 4 | -0.07 | 0.33 |
| 5 | 0.71 | 0.01 |
| 6 | 0.00 | -0.05 |
| 7 | 0.72 | 0.10 |
| 8 | 0.91 | 0.10 |
| 9 | 0.80 | 0.76 |
| 10 | 0.64 | 0.03 |
| 11 | 0.43 | 0.37 |
| 12 | 0.80 | 0.07 |
| 13 | 0.71 | 0.10 |
| 14 | 0.60 | -0.35 |
| 15 | 0.61 | 0.13 |
| 16 | 0.91 | 0.01 |
| 17 | 0.70 | -0.02 |
| 18 | 0.01 | 0.15 |
| 19 | 0.65 | 0.21 |
| 20 | 0.29 | 0.07 |
| **mean** | **0.52** | **0.11** |

***Table S2:*** *Quality of behavioral fits for the computational models of the learning and the gamble task in experiment 2. Pseudo-R^2^ is defined as (r-l)/r where l and r are, respectively, the log likelihoods of the data under the model and under purely random choices (0.5 for every trial).*

| subject | Learning task: pseudo R^2^ | Gamble task: pseudo R^2^ |
| --- | --- | --- |
| 1 | 0.73 | 0.10 |
| 2 | 0.61 | 0.09 |
| 3 | 0.74 | 0.07 |
| 4 | 0.95 | 0.01 |
| 5 | 0.68 | 0.18 |
| 6 | 0.49 | 0.10 |
| 7 | 0.85 | -0.06 |
| 8 | 0.69 | 0.13 |
| 9 | 0.05 | 0.00 |
| 10 | 0.58 | 0.09 |
| 11 | 0.41 | 0.01 |
| 12 | 0.74 | 0.03 |
| 13 | 0.78 | 0.06 |
| 14 | 0.83 | 0.03 |
| **mean** | **0.6520** | **0.0592** |

***Table S3:*** *Quality of behavioral fits for the computational models of the learning and the gamble task in experiment 2. Pseudo-R^2^ is defined as (r-l)/r where l and r are, respectively, the log likelihoods of the data under the model and under purely random choices (0.5 for every trial).*

| subject | Learning task: pseudo R^2^ | Gamble task: pseudo R^2^ |
| --- | --- | --- |
| 1 | 0.71 | 0.08 |
| 2 | 0.77 | 0.15 |
| 3 | 0.67 | 0.10 |
| 4 | 0.36 | 0.02 |
| 5 | 0.77 | 0.01 |
| 6 | 0.79 | 0.01 |
| 7 | 0.80 | 0.01 |
| 8 | 0.49 | 0.36 |
| 9 | -0.20 | 0.09 |
| 10 | 0.64 | 0.15 |
| 11 | 0.76 | 0.10 |
| 12 | -0.01 | 0.01 |
| 13 | 0.09 | 0.12 |
| 14 | 0.68 | -0.01 |
| 15 | 0.80 | 0.02 |
| 16 | 0.42 | 0.01 |
| 17 | 0.55 | 0.01 |
| 18 | 0.34 | -0.02 |
| 19 | 0.95 | 0.17 |
| 20 | 0.65 | 0.01 |
| **mean** | **0.55** | **0.0701** |

**Table S4: Brain areas active in the interaction contrast [(Sure/CSwin + Gamble/CSlose) – (Gamble/CSwin + Sure/CSlose)]. Data is thresholded at p<0.001 uncorrected for multiple comparisons.**

| **structure** | **hemisphere** | **cluster size** | **T** | **z** | **uncorrected p** | **x** | **y** | **z** |
| --- | --- | --- | --- | --- | --- | --- | --- | --- |
| Medial Cingulate Gyrus | L | 52 | 4.54 | 3.69 | 0 | -6 | -6 | 52 |
| Supplementary Motor Area | R |  | 2.89 | 2.6 | 0.005 | 8 | -10 | 54 |
| Medial Cingulate Gyrus | L | 16 | 4.3 | 3.55 | 0 | -16 | -34 | 42 |
| Medial Cingulate Gyrus | L |  | 3.22 | 2.84 | 0.002 | -12 | -34 | 50 |
| White Temporal Lobe | R | 29 | 4.15 | 3.46 | 0 | 38 | -16 | -8 |
| Caudate | R | 45 | 3.96 | 3.34 | 0 | 12 | 22 | 0 |
| Amygdala | L | 22 | 3.88 | 3.29 | 0 | -22 | 6 | -18 |
| Cerebelum | L | 12 | 3.85 | 3.27 | 0.001 | -6 | -40 | -18 |
| Temporal Pole Medial | R | 12 | 3.84 | 3.26 | 0.001 | 36 | 8 | -30 |
| White Occipital Lobe | L | 23 | 3.78 | 3.22 | 0.001 | -32 | -74 | 8 |
| Paracentral | L | 15 | 3.77 | 3.22 | 0.001 | -10 | -22 | 56 |
| Thalamus | R | 24 | 3.73 | 3.19 | 0.001 | 16 | -8 | -2 |
| Parahippocampal gyrus | R | 13 | 3.7 | 3.17 | 0.001 | 24 | -6 | -28 |
| Anterior Cingulate gyrus | L | 10 | 3.63 | 3.12 | 0.001 | -8 | 32 | 28 |
| Cerebelum | L | 28 | 3.62 | 3.11 | 0.001 | -6 | -64 | -28 |
|  |  |  | 3.27 | 2.88 | 0.002 | -8 | -60 | -20 |
| Anterior Cingulate gyrus | R | 73 | 3.59 | 3.1 | 0.001 | 14 | 36 | 30 |
|  |  |  | 3.43 | 2.99 | 0.001 | 8 | 32 | 22 |
|  |  |  | 3.22 | 2.84 | 0.002 | 16 | 34 | 20 |
| Insula | R | 14 | 3.48 | 3.02 | 0.001 | 50 | 12 | -8 |
| Medial Cingulate Gyrus | R | 14 | 3.47 | 3.02 | 0.001 | 4 | 4 | 46 |
| Thalamus | R | 12 | 3.45 | 3 | 0.001 | 10 | -22 | 12 |
| White Cerebelum | R | 10 | 3.41 | 2.98 | 0.001 | 34 | -50 | -38 |
| White Cerebelum | L | 11 | 3.31 | 2.9 | 0.002 | -26 | -52 | -38 |
| Lingual gyrus | R | 13 | 3.28 | 2.88 | 0.002 | 2 | -72 | 4 |
| Insula | R | 12 | 3.23 | 2.85 | 0.002 | 50 | 2 | 0 |
| Medial Temporal gyrus | L | 20 | 3.16 | 2.8 | 0.003 | -2 | -50 | -50 |
